# Perspectives of the AMP-activated kinase (AMPK) signalling pathway in thyroid cancer

**DOI:** 10.1042/BSR20130134

**Published:** 2014-04-15

**Authors:** Bruno Moulin Andrade, Denise Pires de Carvalho

**Affiliations:** Laboratório de Fisiologia Endócrina, Instituto de Biofísica Carlos Chagas Filho, Universidade Federal do Rio de Janeiro, Rio de Janeiro, Brazil

**Keywords:** AMPK, metformin, mTOR, NIS, thyroid cancer, Warburg effect, ACC, acetyl-CoA-carboxylase, AICAR, 5-amino-4-imidazolecarboxamide riboside, AMPK, AMP-activated protein kinase, CTC, circulating tumour cell, DTC, differentiated thyroid carcinomas, ERK, extracellular-signal-regulated kinase, ^18^F-FDG-PET, ^18^F-fluoro-deoxiglucose positron emission tomography, GLUT, glucose transporters, GLUT1, glucose uptake and glucose transporter 1, HIF-1, hypoxia-inducible factor 1, LKB1, liver kinase B1, mTOR, mammalian target of rapamycin, mTORC1, mammalian target-of-rapamycin complex-1, NIS, Na^+^/I^−^-symporter, p70S6K, p70 S6 kinase, PI3K, phosphoinositide 3-kinase, PTC, papillary thyroid cancer, PTEN, phosphotyrosine phosphatase, RAS, renin–angiotensin system, ROS, reactive oxygen species, TCA, tricarboxylic acid, TSH, thyroid-stimulating hormone

## Abstract

Approximately 90% of non-medullary thyroid malignancies originate from the follicular cell and are classified as papillary or follicular (well-differentiated) thyroid carcinomas, showing an overall favourable prognosis. However, recurrence or persistence of the disease occurs in some cases associated with the presence of loco-regional or distant metastatic lesions that generally become resistant to radioiodine therapy, while glucose uptake and metabolism are increased. Recent advances in the field of tumor progression have shown that CTC (circulating tumour cells) are metabolic and genetically heterogeneous. There is now special interest in unravelling the mechanisms that allow the reminiscence of dormant tumour lesions that might be related to late disease progression and increased risk of recurrence. AMPK (AMP-activated protein kinase) is activated by the depletion in cellular energy levels and allows adaptive changes in cell metabolism that are fundamental for cell survival in a stressful environment; nevertheless, the activation of this kinase also decreases cell proliferation rate and induces tumour cell apoptosis. In the thyroid field, AMPK emerged as a novel important intracellular pathway, since it regulates both iodide and glucose uptakes in normal thyroid cells. Furthermore, it has recently been demonstrated that the AMPK pathway is highly activated in papillary thyroid carcinomas, although the clinical significance of these findings remains elusive. Herein we review the current knowledge about the role of AMPK activation in thyroid physiology and pathophysiology, with special focus on thyroid cancer.

## Abstract 2

Recurrence or persistence of differentiated thyroid cancer can be associated with resistance to radioiodine therapy, which is associated with higher glucose uptake and metabolism by the remnant lesions. The possible role of AMPK activation in this phenomenon is discussed.

## INTRODUCTION

Palpable and non-palpable thyroid nodules are common endocrine tumours that can be detected in as much as 70% of a population if sensitive image-assisted examination methods are used [1]. Independent variables as gender and age influence the prevalence of these nodules, with a higher risk for women and elderly individuals [2].

Although nodular lesions are frequent in the overall population, only 5–10% of thyroid nodules correspond to malignant lesions, and thyroid carcinoma is a rare disease that accounts for just 1% of all human cancers, although it is the most common endocrine malignancy [1,3]. Approximately 90% of non-medullary thyroid malignancies originate from the follicular cell and are classified as papillary or follicular (well-differentiated) thyroid carcinomas. DTC (differentiated thyroid carcinomas) are slow growing tumours that can be curable by the combined effects of surgery, radioiodine ablation and TSH (thyroid-stimulating hormone) suppressive therapy. However, during tumour progression that occurs in up to 20–30% of cases, cellular dedifferentiation is present, and is usually accompanied by more aggressive growth, metastatic spread and loss of iodide uptake ability [4].

Four types of genetic alterations comprise the majority of known mutations in DTC: BRAF and RAS (renin–angiotensin system) point mutations, and RET/PTC rearrangements for papillary thyroid cancers, and PAX8/PPARγ rearrangement in the follicular subtype [5]. The incidence of thyroid cancer has increased in the past decades in many countries, including Brazil [6–11], and this increase is mainly due to the rise in the incidence of PTC. Jung et al. [12] recently reported that in the USA the overall proportion of BRAF mutations remained stable, the percentage of RET/PTC rearrangements decreased, while there seems to be a rise of the follicular variant histology subtype and RAS mutations incidence after 2000.

The adequate intervention for DTC vary depending on the disease stage, rather than the different causative mutations. In the past 10 years, risk stratification systems were reported in order to try to better establish the adequate intervention for each patient [13,14]. In this context, the serum levels of thyroglobulin, anti-thyroglobulin and the identification of structural disease by traditional tomography, scintigraphy with ^131^I-radioiodide and ^18^F-FDG-PET (^18^F-fluoro-deoxiglucose positron emission tomography) are very important criteria for assessment of recurrent or persistent disease. Indeed, the increase of ^18^F-FDG uptake associated with a decrease in iodide uptake indicates poor prognostics [13–15]. These image scans depend on the cellular ability to uptake either iodide or glucose. Iodide uptake by follicular thyroid cells is mediated by a transmembrane glycoprotein known as the NIS (Na^+^/I^−^-symporter), which is a thyrocyte differentiation marker that is lost in the course of thyroid carcinogenesis [14,16,17]. On the other hand, glucose uptake depends on the presence of GLUTs (glucose transporters). As a result, there is a great interest of a better understanding about the regulation of these transporter systems.

Although, iodide uptake through NIS is involved in both diagnostic and treatment of thyroid cancer, the mechanism underlying NIS expression and subcellular localization in thyroid cells has not been completely elucidated. Also, the molecular mechanisms involved in the metabolic shift that leads to higher glucose uptake by tumour cells are poorly defined. Recently, we described that the energy sensor AMPK (AMP-activated protein kinase) plays an important physiological role in the thyroid gland by regulating both iodide and glucose uptakes [18,19]. In fact, AMPK activation in the normal thyrocyte induces a dramatic reduction of iodide uptake that is accompanied by higher glucose uptake and utilization by the glycolytic pathway. Until now, only a few studies analysed the AMPK pathway in thyroid cells, and a recent report shows that the metformin treatment induces thyroid tumour cell apoptosis [20]. On the other hand, we have recently shown that the expression and activity of AMPK is increased in papillary thyroid carcinoma [21].

In this review, we focused on the current knowledge about NIS regulation by AMPK in thyroid cells and the possible involvement of AMPK signalling pathway in thyroid cancer cell biology.

## AMPK STRUCTURE AND METABOLIC FUNCTION

**Figure 1:**
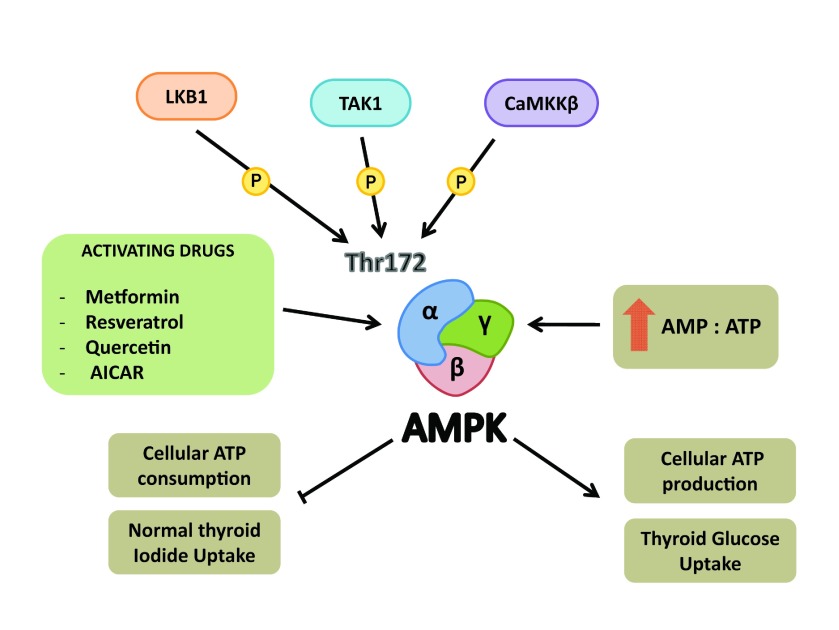
Schematic representation of AMPK subunits α,β,γ Allosteric activation of AMPK occurs through an increase in the intracellular AMP/ATP ratio that allows upstream kinases such as LKB1 (liver kinase B1), TAK1 (TGF-β-activated kinase 1) or CAMKKβ (Calmodulin kinase kinase β) to phosphorylate Thr^172^ in AMPK α-catalytic subunit. Some drugs are also able to directly or indirectly activate AMPK. Once activated, AMPK inhibits ATP-consuming process and stimulates ATP synthesis pathways. In the normal thyroid, iodide uptake decreases and glucose uptake increases by AMPK activation.

AMPK is a metabolic stress-sensing cytosolic enzyme, composed of an α-catalytic and two regulatory (β and γ) subunits [22]. Stresses that deplete cell energy and increase the intracellular AMP-to-ATP ratio induce allosteric activation of AMPK, promoting conformational changes that make the enzyme a substrate for upstream kinases (Figure 1) that phosphorylate its threonine-172 residue and activate AMPK [22,23]. When activated, AMPK shuts down cell energy consumption and up-regulates processes that increase energy production, in an attempt to restore intracellular ATP levels [23]. Indeed, one of the well-described effects of AMPK is the inhibition of ACC (acetyl-CoA-carboxylase) enzyme through the phosphorylation of its serine 79. ACC is responsible for the conversion of acetyl-CoA to malonyl-CoA in *de novo* lipid biosynthesis. In turn, manolyl-CoA is a potent inhibitor of carnitine palmitoyl transferase-1, responsible for the translocation of long-chain fat acid into mitochondrial matrix. The reduction of malonyl-CoA induced by AMPK activation is believed to favour fat acid translocation into the mitochondria and β-oxidation to increase ATP production [22–25].

## AMPK AND THE THYROID GLAND

Our group was the first to describe the expression and function of AMPK in the thyroid gland, and its ability to regulate iodide and glucose uptakes in thyroid cells [18,19]. Indeed, the pharmacologic activator of AICAR (AMPK, 5-amino-4-imidazolecarboxamide riboside), decreased sodium-iodide symporter expression both at the transcriptional and translational levels. Moreover, the main stimulator of thyroid gland function, the thyrotrophic hormone (TSH) inhibits AMPK phosphorylation and activation [18]. Thus, the pharmacological activation of AMPK in normal thyrocytes results in decreased iodide uptake counterbalancing TSH action [18]. We further demonstrated that AICAR treatment increased GLUT1 (glucose uptake and glucose transporter 1) protein expression in thyroid cells [19]. Based on these results, we hypothesized that AMPK activation could be a common pathway involved in the phenomenon that occurs during thyroid tumour progression, when increased glucose uptake detected by ^18^F-FDG-PET is accompanied by decreased radioiodide uptake ability in DTC [14,15]. However, in a LKB1 (liver kinase B1)- and AMPK-deficient mouse model of Peutz-Jeghers syndrome, there is up-regulation of mTOR (mammalian target of rapamycin) and HIF-1 (hypoxia-inducible factor 1) α transcription factor that leads to higher hexokinase II and Glut1 expression and increased glucose utilization by the tumours [26]. Thus, the interplay between mTOR, AMPK and HIF-1α in the thyroid remains to be elucidated.

More recently, Abdulrahman et al. [27] confirmed our results in rat thyrocytes, using metformin that indirectly activates AMPK. Interestingly, this study showed that AMPK-α1 knockout mice have a less active thyroid gland with reduced responsiveness to TSH and reduced colloid volume. All these recent findings demonstrated that AMPK is not only expressed in the thyroid gland but that it also plays a physiological role in the thyrocyte.

## AMPK SIGNALLING AND CANCER

The evidence that the tumour suppressor LKB1 is the major upstream kinase responsible for AMP activation linked for the first time AMPK activity and cancer development [24]. Since LKB1 loss-of-function mutations are related to cancer development, AMPK was largely believed to be down-regulated in cancer cells. However, a large controversy exists in the literature in relation to the AMPK pathway involvement in tumourigenesis and cancer progression.

Several studies demonstrated that activated AMPK causes cell-cycle arrest associated with stabilization of p53 and decreased protein and lipid synthesis and ribosomal RNA [20,28]. Indeed, AICAR-mediated AMPK activation has a strong anti-proliferative effect in different cancer cell lines [20] and in human breast cancer samples [29]. Also, AMPK activation shuts down processes that consume energy through modulation of several signalling pathways. One of the most described downstream targets of AMPK is the inhibition of the serine–threonine protein kinase mTORC1 (mammalian target-of-rapamycin complex-1) by phosphorylating its upstream regulator TSC2 (tuberous sclerosis complex 2) and its regulatory subunit Raptor [24,25,30]. Since the mTOR/p70S6K (p70 S6 kinase) pathway stimulates cell proliferation and metabolism, the AMPK-mediated inhibition of this pathway corresponds to an important strategy to restore ATP levels [23–25,30]. These findings are in agreement with the hypothesis that AMPK inhibition might lead to higher cell proliferative rates.

Altogether, these previously described AMPK effects are mainly anti-proliferative and anti-tumourigenic. However, different studies demonstrated that AMPK activation mediated by the reduction of ATP/AMP ratio promotes cellular survival under stressful metabolic conditions that are characteristic of tumour microenvironment [22,25,31]. Indeed, cancer cells have profound alterations in their metabolism that are fundamental for survival in tumour microenvironment and metastatic niche. Tumour cells are largely dependent on the biosynthesis of macromolecules and the tightened maintenance of appropriate cellular redox status [25]. The idea that AMPK activation should in turn allow cell survival and quiescence under unfavourable conditions have been supported by Buzzai et al. [31] who demonstrated that AMPK activation is capable to revert cell death promoted by glucose deprivation of Akt-expressing glioblastoma cells. Although the intracellular pathway triggered by AMPK to impair cell death has not been completely elucidated, the involvement of HIF-1 and maintenance of cellular NADPH homoeostasis have been proposed [22,32]. Although some evidence shows that AMPK-deficient fibroblasts have increased levels of HIF-1α and its downstream targets, other studies have asserted that AMPK activation up-regulates HIF-1 and VEGF (vascular endothelial growth factor) expression under hypoxic conditions [26,33]. Some authors claim that the expression of HIF1α and HIF2α are associated with a poor prognosis in thyroid tumours [34,35], but more studies are necessary to clarify whether these findings are related to AMPK regulation in thyroid cancer.

Recently, the participation of ROS (reactive oxygen species)/AMPK activation as fundamental for the Warburg effect in cancer cells has been confirmed [36]. In fact, nutrient deficiency increases ROS production that in turn activates AMPK that triggers pyruvate dehydrogenase kinase activation and pyruvate dehydrogenase phosphorylation. This pathway seems to be essential to drive cancer cells to increase glycolysis to produce ATP, even in aerobic condition.

Recent advances in the field of tumour progression have shown that CTC (circulating tumour cells) are metabolic and genetically heterogeneous [37]. Based on these findings, it is tempting to speculate which are the characteristics of the tumour cell that are important for cancer dissemination and increased risk of recurrence. Surely, CTC are capable of surviving in unfavourable conditions and the metastatic niche development might depend on these special features.

## METFORMIN, AMPK AND THYROID NODULES IN DIABETIC PATIENTS

The strikingly findings that type 2 diabetic patients have increased incidence of thyroid nodules and thyroid volume [38] are under current investigation by several other groups. Also, insulin resistance and hyperinsulinaemia were identified as risk factors for developing DTC [39].

Subsequent reports described that metformin, a drug used for the management of type 2 diabetes, might present antineoplastic effects in various human cancers [40,41]. Taking into consideration the mechanism of action of the antidiabetic drug metformin, the AMPK-signalling pathway has become an interesting target in thyroid cancer studies. In the intracellular level, metformin inhibits mitochondrial complex I, which leads to an altered AMP/ATP ratio and the activation of AMPK [40,42]. Apart from the AMP/ATP ratio disturbance, metformin might also change mitochondria ROS production and thus secondarily promote AMPK activation [43]. It is now believed that the long-term activation of AMPK by increased ROS generation in cancer cells leads to mTORC1 complex suppression, resulting in cell death, which was demonstrated for sorafenib effects on cell energy metabolism [44].

Studies on various cancer cell lines and animal models demonstrated that metformin inhibits tumour growth via the activation of AMPK and the inhibition of mTOR/S6K (S6 kinase) signalling pathway [41,45,46]. It is noteworthy that these studies report the association between cancer incidence in diabetics treated with metformin compared with those using other medications (typically sulphonylureas or insulin), and this evidence is so far not enough to prove a causal link.

## AMPK AND THYROID CANCER

The AMPK pathway has not been extensively studied in thyroid cancer and until recently the expression and function of AMPK in this type of cancer was not evaluated so far. In a recent report, AMPK expression was first described in the human thyroid gland and both its expression and activity were shown to be strongly up-regulated in PTC [21]. Using a TMA (tumour micro array) slide, total and phosphorylated AMPK were analysed by immunohistochemistry, as well as phosphorylated ACC expression in normal and PTC samples from the same patients. Interestingly, AMPK pathway activation did not occur in adenomas when compared with the non-neoplastic tissue from the same patients. More data are now required to give us a comprehensive understanding about the role of AMPK pathway in differentiated and undifferentiated thyroid carcinomas, and its role in carcinogenesis.

Using thyroid cancer cell line expressing either wild-type BRAF or V600E-mutant BRAF, Choi et al. [47] observed that AICAR treatment induced a decrease in the cell proliferation rate associated with increased S-phase cell-cycle arrest and apoptosis. Interestingly, AMPK suppressed the phosphorylation of ERK (extracellular-signal-regulated kinase) and p70S6K (mTOR target), in BRAF V600E mutant thyroid cancer cells, but rather increased their phosphorylation in wild-type cells [47]. These previous results confirm the anti-proliferative effect of AMPK in thyroid cancer cells, as also demonstrated for other carcinomas. However, based on the well-described negative effect of AMPK on the mTOR pathway in other tissues, it is tempting to speculate whether AMPK activation also promote mTORC1 inhibition in normal thyroid cells, as well in the different tumours derived from the thyroid follicular cell.

The mTOR pathway is also up-regulated in DTC [48], and mTOR inhibition by rapamycin increases normal rat thyroid cell iodide uptake [49]. Although the interrelationship between AMPK and mTOR is well described in metabolic tissues such as skeletal muscle, liver and adipose tissue, the AMPK ability to inhibit mTOR has still to be demonstrated in thyroid cell physiology and thyroid cancer. Taking into consideration that in the normal thyroid both the AMPK [18] and the mTOR [49] pathways inhibit iodide uptake, one can speculate that no matter the balance between these two pathways, just the activation of one of them can explain the decrease of iodide uptake. The same rational is true regarding the glucose uptake ability, since mTOR increases GLUT1 expression via HIF1α in some tumours [26], while AMPK pathway also leads to increased glucose uptake and hexokinase activity in normal thyrocytes [19].

Recently, Antico Arciuch et al. [50] described an elegant model of tumourigenesis using mice with thyroid-specific PTEN (phosphotyrosine phosphatase) deficiency. The thyroids of PTEN-deficient animals have constitutively activated the PI3K (phosphoinositide 3-kinase) pathway, leading to hyperplastic thyroid glands at birth and the development of thyroid follicular adenomas and metastatic follicular carcinomas late in adult life. Constitutive PI3K activation initiates the remodelling of cell energy metabolism through a decrease in the expression of the enzymes of the TCA (tricarboxylic acid) cycle, with reduced mitochondria respiratory capacity and increased metabolic flux through glycolysis, as indicated by the dramatic increase in lactate production. Interestingly, *Pten*^thy−/−^ mice had a strong decreased in AMPK activity (AMPK phophorylation on Thr^172^), mainly through PI3K and PKA-mediated AMPK phosphorylation at Ser^485^. In order to reactivated AMPK, they treated the mutants with AICAR for 4 weeks. They observed a restoration in the expression of TCA enzymes and a reversion in the glycolytic switch induced by constitutive PI3K activation. Also, AMPK reactivation led to a slower growth rate in mutant glands compared with untreated mutants, which was confirmed by a drastically reduced thyroid BrdUrd (bromodeoxyuridine) incorporation in AICAR treated, when compared with that of untreated mice. These findings are in agreement with the tumour suppressor role of AMPK, but are not fully concordant with the finding of increased AMPK signalling pathway that was detected in human thyroid cancer samples.

Metformin treatment inhibited growth and induced apoptosis in anaplastic thyroid cancer cell lines [51]. Furthermore, Klubo-Gwiezdzinska et al. [52] showed that metformin also inhibited growth of medullary thyroid cancer cells in a dose- and time-dependent manner, with decreased expression of cyclin D1. In an attempt to investigate the signalling pathway that mediates these effects of metformin in medullary cancer cells, the authors found an inhibition of mTOR/p70S6K/pS6 signalling and the down-regulation of pERK. The authors also showed that eight out of the 14 (57%) human medullary tumours analysed had increased phospho-p70S6K expression compared with the corresponding normal thyroid tissue. However, the treatment with AMPK inhibitor (compound C) or AMPK silencing did not prevent growth inhibitory effects of metformin in medullary cancer cells, which suggest that metformin modulates different pathways besides AMPK signalling to induce its anti-proliferative effects. More recently, the same group demonstrated that diabetic patients that were treated with metformin for more than 4 years had significantly smaller thyroid tumour size then diabetic and non-diabetic patients not treated with metformin. These authors demonstrate that tumours from patients treated with metformin show a lower level of phospho-p70S6K compared with the non-treated diabetic group [53].

The new findings of higher AMPK activation in human papillary thyroid cancer in relation to benign lesions and the possibility that this pathway modulate cell growth, apoptosis and survival raises several questions that need to be answered in order to better define whether AMPK could be a novel target in thyroid cancer patients. The differentiated papillary thyroid cancer is indolent and most of these tumours do not present an aggressive behaviour. It is intriguing that this same tumour subtype seems to express high levels of both mTOR [47] and AMPK [21] signalling pathways. The most important question is whether the over activation of these proteins is only implicated in tumour cell survival under stressful conditions, or they are fundamental for tumour growth and invasion. Thus, it is now important to better define which is the interplay between these two pathways during tumour progression and metastatic spread.

## FINAL REMARKS

AMPK functions as a tumour suppressor gene in several cancer subtypes, and accordingly its activation in tumour cells lead to cell death. As shown, AMPK activation in normal cells or its loss of function in tumours can also be the molecular pathway implicated in the higher glucose uptake that occurs during the tumour metabolic shift known as the Warburg effect. Thus, it is of great importance to better understand the pleiotropic actions of the different AMPK isoforms, their levels of activation and their perspectives in cancer cell biology. Since AMPK pathway is overexpressed in primary differentiated tumours of the human thyroid, it is now intriguing to know what are the consequences for tumour cells of its further activation or rather silencing. Also, the study of AMPK pathway in advanced and the undifferentiated anaplastic thyroid cancer can bring further insight into the role of AMPK in the different stages of cancer progression.
